# Baseline Chronic Comorbidity and Mortality in Laboratory-Confirmed COVID-19 Cases: Results from the PRECOVID Study in Spain

**DOI:** 10.3390/ijerph17145171

**Published:** 2020-07-17

**Authors:** Beatriz Poblador-Plou, Jonás Carmona-Pírez, Ignatios Ioakeim-Skoufa, Antonio Poncel-Falcó, Kevin Bliek-Bueno, Mabel Cano-del Pozo, Luis Andrés Gimeno-Feliú, Francisca González-Rubio, Mercedes Aza-Pascual-Salcedo, Ana Cristina Bandrés-Liso, Jesús Díez-Manglano, Javier Marta-Moreno, Sara Mucherino, Antonio Gimeno-Miguel, Alexandra Prados-Torres, EpiChron Group

**Affiliations:** 1 EpiChron Research Group, Aragon Health Sciences Institute (IACS), IIS Aragón, Miguel Servet University Hospital, 50009 Zaragoza, Spain; bpoblador.iacs@aragon.es (B.P.-P.); jcarmona@iisaragon.es (J.C.-P.); ignacio.ioakim@hotmail.es (I.I.-S.); aponcel@salud.aragon.es (A.P.-F.); micano@aragon.es (M.C.-d.P.); lugifel@gmail.com (L.A.G.-F.); franciscagonzalezrubio@gmail.com (F.G.-R.); maza@salud.aragon.es (M.A.-P.-S.); acbandres@salud.aragon.es (A.C.B.-L.); jdiez@aragon.es (J.D.-M.); jmartam@gmail.com (J.M.-M.); sprados.iacs@aragon.es (A.P.-T.); 2 Health Services Research on Chronic Patients Network (REDISSEC), ISCIII, 28222 Madrid, Spain; 3 Delicias-Sur Primary Care Health Centre, Aragon Health Service (SALUD), 50009 Zaragoza, Spain; 4 Aragon Health Service (SALUD), 50017 Zaragoza, Spain; 5 Teaching Unit of Preventive Medicine and Public Health, Miguel Servet University Hospital, 50009 Zaragoza, Spain; 6 General Directorate of Health Care, Department of Health, Government of Aragon, 50017 Zaragoza, Spain; 7 San Pablo Primary Care Health Centre, Aragon Health Service (SALUD), University of Zaragoza, 50003 Zaragoza, Spain; 8 Primary Care Pharmacy Service Zaragoza III, Aragon Health Service (SALUD), 50017 Zaragoza, Spain; 9 Service of Internal Medicine, Royo Villanova Hospital, Aragon Health Service (SALUD), 50015 Zaragoza, Spain; 10 Service of Neurology, Miguel Servet University Hospital, Aragon Health Service (SALUD), 50009 Zaragoza, Spain; 11 CIRFF, Center of Pharmacoeconomics, Department of Pharmacy, University of Naples Federico II, 80138 Napoli, Italy; sara.mucherino@unina.it

**Keywords:** chronic diseases, cohort, comorbidity, COVID-19, drugs, gender, mortality, multimorbidity, Spain

## Abstract

We aimed to analyze baseline socio-demographic and clinical factors associated with an increased likelihood of mortality in men and women with coronavirus disease (COVID-19). We conducted a retrospective cohort study (PRECOVID Study) on all 4412 individuals with laboratory-confirmed COVID-19 in Aragon, Spain, and followed them for at least 30 days from cohort entry. We described the socio-demographic and clinical characteristics of all patients of the cohort. Age-adjusted logistic regressions models were performed to analyze the likelihood of mortality based on demographic and clinical variables. All analyses were stratified by sex. Old age, specific diseases such as diabetes, acute myocardial infarction, or congestive heart failure, and dispensation of drugs like vasodilators, antipsychotics, and potassium-sparing agents were associated with an increased likelihood of mortality. Our findings suggest that specific comorbidities, mainly of cardiovascular nature, and medications at the time of infection could explain around one quarter of the mortality in COVID-19 disease, and that women and men probably share similar but not identical risk factors. Nonetheless, the great part of mortality seems to be explained by other patient- and/or health-system-related factors. More research is needed in this field to provide the necessary evidence for the development of early identification strategies for patients at higher risk of adverse outcomes.

## 1. Introduction

Six months after the first case of infection by severe acute respiratory syndrome coronavirus 2 (SARS-CoV-2) reported in Wuhan, China, in December 2019, the virus has spread worldwide [1,2]. The World Health Organization has declared that the ongoing pandemic of coronavirus disease 2019 (COVID-19) represents a public health emergency of international concern. Even though governments all around the world have adopted control measures, mainly through social distancing and isolation of patients, the coronavirus outbreak has affected over 200 countries with approximately 8 million confirmed cases and over 445,000 deaths worldwide as of 1 June 2020 [3]. Spain was the second country in Europe after Italy to suffer the COVID-19 pandemic, with the first case reported in January 2020, and is one of the countries with the highest number of attributable deaths after the United Kingdom, Italy and France at the moment of writing this study [3].

The impact of the disease has varied among countries, sub-populations and ethnicities in terms of fatality rates and other health-related outcomes, which could be explained not only by genetic differences but also by diverse cultural, political, and socio-behavioral factors [4,5]. Its rapid geographic spread across countries requires large-scale epidemiological studies to ascertain and develop effective health policies considering the general context in which the outbreak has taken place.

The first published reports of COVID-19 were mostly case series of hospitalized patients, describing the more severe end of the clinical spectrum of the disease [6], mainly life-threatening pneumonia. Later observational studies reported a wide range of symptoms, from asymptomatic infection to respiratory failure and death [7]. It has been estimated that eight out of every ten patients infected with SARS-CoV-2 will develop mild symptoms, while the remaining two can evolve to severe or critical conditions [8].

As with other acute respiratory infections, certain comorbidity profiles could predispose COVID-19 patients to more frequent adverse outcomes. Several chronic conditions, such as diabetes, hypertension, cardiovascular disease, cerebrovascular disease, or chronic obstructive pulmonary disease have already been identified [9,10], while other factors, such as older age or d-dimer levels on admission over 1 μg/mL in hospitalized patients, have also been found to entail poorer health outcomes [8,11]. However, previous reports on the role that other baseline chronic conditions and medications play in the evolution and outcomes of infected patients are contradictory, and the relevance of these factors remains unclear.

Longitudinal, population-based epidemiological studies, in both inpatient and outpatient settings, including baseline information on every possible disease and medication, are necessary to shed light on the risk factors behind the most severe cases. The results of such an approach are relevant for the early identification of patients susceptible to poorer health outcomes and, therefore, potential targets of specific prevention strategies and/or of closer clinical monitoring.

This exploratory study aims to analyze which socio-demographic and clinical factors are associated with an increased risk of mortality in a Spanish cohort of individuals infected by COVID-19, with a particular focus on baseline chronic diseases and treatments present at the time of infection.

## 2. Materials and Methods

### 2.1. Design and Study Population

We conducted a retrospective cohort study (PRECOVID Study), including all individuals with laboratory-confirmed infection by SARS-CoV-2 in the Spanish region of Aragon (reference population, 1.3 million inhabitants), from a total of 14,724 laboratory-tested individuals. In Aragon, the national health system provides free-of-charge healthcare with universal coverage to approximately 95% of the population. The PRECOVID Study was created to serve as the basis for future observational studies on the epidemiology and trajectory of the disease through the linkage of real-world data from patients’ electronic health records (EHRs) and clinical-administrative databases.

For this study, we included all the individuals of the cohort from 4 March 2020 (i.e., date of the first confirmed infection in the region) to 17 April 2020 (enrolment period). We followed patients from the date of inclusion in the cohort to 17 May 2020, or to the date of death, to ensure a minimum follow-up period of 30 days for all analyzed patients and allow sufficient time for the studied event (i.e., death) to occur. Laboratory tests were conducted in the region to confirm the infection in hospitalized patients with compatible symptoms, exposed healthcare workers, residents and workers from social care institutions where an outbreak was taking place (e.g., residential homes for the elderly; physical or mental disability care centers), and individuals at a community level associated to family or work-related clusters of cases.

This study was conducted in line with the Declaration of Helsinki, as revised in 2013. The Clinical Research Ethics Committee of Aragón (CEICA) approved the research protocol (PI20/226). CEICA waived the requirement to obtain informed consent from patients given the epidemiological nature of the project, which used anonymized data.

### 2.2. Variables of Study and Data Sources

For each individual of the cohort, we analyzed the following baseline variables at the time of inclusion: (i) socio-demographic characteristics: Sex, age (0–14, 15–44, 45–64, 65–79, ≥80 years), country of birth (native vs. non-native), administrative health area (urban vs. rural), and deprivation index of the area measured at an aggregated level and divided into four quartiles from least (Q1) to most (Q4) deprived [12]; and (ii) clinical information: All chronic disease diagnoses present at the date of infection and registered in primary care EHRs, all drugs dispensed during the month prior to infection in community and hospital pharmacies, presence of multimorbidity (i.e., the coexistence of two or more chronic conditions) and polypharmacy (i.e., the dispensation of five or more drugs), Anticholinergic Drug Scale (ADS) score [13] based on an updated version [14], and flu vaccination. During the follow-up period, we analyzed all-cause mortality. Hospitalizations and Intensive Care Unit admissions were not analyzed due to potential biases influenced by the healthcare system´s organization, amongst others.

Primary care diagnoses were initially coded using the International Classification of Primary Care, First Edition (ICPC–1). These codes were mapped to ICD-9-CM using a mapping system developed to codify temporary disability [15]. Diagnoses were subsequently sorted into 226 clinically relevant categories using the Clinical Classifications Software (CCS) for ICD-9-CM [16]. Diagnoses were classified as chronic or not chronic using the Chronic Condition Indicator software tool [17], with a total of 153 conditions categorized as chronic. Some of the diagnostic labels were renamed to facilitate their clinical interpretation. Chronic conditions were defined as those present during the last 12 months at least, and meeting one or both of the following criteria: (i) Entails limitations on self-care, independent living, and social interactions; (ii) requires of ongoing interventions using medical products, services, and special equipment. We analyzed all drugs using their Anatomical-Therapeutic-Chemical (ATC) classification system code at the third level (i.e., pharmacological subgroup). The ADS score was rated from level 0 (absence of known anticholinergic properties) to level 3 (markedly anticholinergic), as previously described [13,14].

Patient data was obtained from the Aragon Health System linking, at a patient level and in a pseudo-anonymized form, the information contained in the users’ database, primary care EHRs, primary care and hospital pharmaceutical billing records, and an *ad hoc* registry developed for monitoring the evolution of the COVID-19 disease pandemic in the region of Aragon.

### 2.3. Statistical Analysis

We described the socio-demographic and clinical characteristics of all patients from the cohort (alive and deceased at the end of follow-up) as means accompanied by their respective standard deviations or as absolute and relative frequencies and proportions, by sex. Differences in means between alive and deceased men and women were analyzed using age-adjusted logistic regression analysis.

We performed logistic regression models to analyze the association between the presence of each chronic comorbidity and drug studied at baseline and the likelihood of mortality during the follow-up period, by sex. We used this technique instead of Cox regression since we had the date of registry but not the exact date of infection and/or of confirmatory test for all patients. We calculated age-adjusted odds ratios (ORs) accompanied by their respective 95% confidence intervals (CI). For this analysis, only chronic diseases and medications with at least 10 cases in deceased men and/or women were considered.

Finally, we performed a multivariate logistic regression analysis to identify which of the studied variables were significantly associated with higher mortality when studied in combination. In doing so, we included as potential explanatory variables all socio-demographic characteristics, chronic diseases and medications that showed significant association at baseline. Multivariate analysis was performed using a stepwise method with an exit *p*-value for analyzed variables of 0.05. We calculated the pseudo R squared statistics to estimate the proportion of the variation in mortality that was explained by the variables included in each model for men and women. Statistical analyses were conducted using Stata software (Version 12.0, StataCorp LLC, College Station, TX, USA), and statistical significance was set at *p* < 0.05. 

## 3. Results

A total of 4412 individuals (58.8% women, mean age of 67.7 (standard deviation, s.d., 20.7) years) were included in the study (Table 1). Of them, 771 (47.2% women, mean age of 84.2 (s.d. 10.0) years) died during the follow-up period. The crude overall mortality rate during the studied period was 3.84 deaths per 100,000 persons per day. Three in four deceased patients were older than 80 years, compared to one in four in those alive. Men died at an earlier age than women (81.5 vs. 87.2 years). The mean numbers of chronic diseases (5.22 vs. 3.03; 6.66 vs. 3.67) and dispensed medications (5.41 vs. 3.00; 5.57 vs. 2.75) were higher in deceased men and women compared to their alive counterparts. Deceased individuals also showed a higher burden of medications with anticholinergic activity, especially women. Although the proportion of influenza-vaccinated individuals was higher in deceased compared to alive men (64.1% vs. 36.3%) and women (68.4% vs. 31.9%), these differences were not significant after controlling for age.

The most prevalent chronic conditions in the individuals of the cohort who died during the follow-up period were hypertension (49.4%), obesity and other nutritional, endocrine and metabolic disorders (28.7%), cataract (28.0%), diabetes (27.5%), and urinary incontinence (25.3%) in men, and hypertension (62.6%), urinary incontinence (50.5%), obesity and other nutritional, endocrine and metabolic disorders (37.9%), mood disorders (36.3%), and osteoarthritis (33.2%) in women (Table 2). These figures were slightly different from those observed in the entire population (see Supplementary Table S1). In men, nine conditions with at least 10 cases amongst deceased individuals were significantly associated with an increased likelihood of death, including (OR (95% CI)) epilepsy (2.56 (1.09–5.99)), inflammatory conditions of genital organs (2.56 (1.15–5.70)), and congestive heart failure (2.15 (1.30–3.54)), among others. Rheumatoid arthritis was also highly associated (5.15 (1.13–23.5)), but only five cases were observed (see Supplementary Table S1). In women, acute myocardial infarction (2.98 (1.25–7.11)), coagulation and hemorrhagic disorders (1.85 (1.15–2.97)), and degenerative nervous system conditions (1.56 (1.05–2.33)) could be highlighted amongst the eight conditions significantly associated with higher mortality. Diabetes (1.65 (1.23–2.21); 1.40 (1.02–1.91)) and chronic ulcer of the skin (2.24 (1.26–3.96); 2.04 (1.35–3.10)) were the only two conditions associated with higher mortality in both sexes.

Some of the most frequently dispensed drugs among individuals who died of both sexes were (men vs. women) drugs for peptic ulcer and gastro-esophageal reflux disease (GORD) (49.6% vs. 58.5%), antithrombotic agents (36.9% vs. 39.0%), other analgesics and antipyretics (31.0% vs. 37.4%), high-ceiling diuretics (24.6% vs. 34.6%), and antidepressants (20.4% vs. 38.7%), among others (Table 3).

The dispensation of 12 drugs in men and 9 drugs in women was associated with an increased likelihood of death during the study period. Out of all of them (OR (95% CI)), drugs for peptic ulcer and GORD (1.45 (1.13–1.86); 1.32 (1.02–1.71)), high-ceiling diuretics (1.79 (1.30–2.49); 1.71 (1.29–2.27)), antipsychotics (1.71 (1.18–2.48); 1.96 (1.41–2.73)), and potassium-sparing agents (2.52 (1.27–4.97); 2.86 (1.50–5.44)) were associated with higher risk of mortality in both men and women, respectively. Women treated with other beta-lactam antibacterials, antigout preparations, and antiepileptics presented 2.17, 2.06, and 1.94 times greater mortality risk than those not receiving these medications, respectively. Men treated with corticosteroids, vasodilators for cardiac diseases, and immunosuppressants showed 3.83, 3.15, and 2.49 times higher risk of death. Plain angiotensin-converting enzyme (ACE) inhibitors were not associated with a higher risk of death in men; however, patients in treatment with medications of ACE inhibitors in combination with other active ingredients were less likely to die (0.46 (0.23–0.93)). Supplementary Table S2 displays the complete list of drugs analyzed in the study population.

The models developed to assess mortality risk revealed that age was the most influencing factor, being the risk of death between 10 and 40 times higher in men and women above 80 years of age compared with their younger 45–64-year-old counterparts (Table 4). Three diseases and six drugs in men, and three diseases and five drugs in women, remained significant in the models. In men, the likelihood of mortality was 2.04, 1.48, and 1.46 times higher when chronic ulcer of skin, cataract, and diabetes were present, respectively. In women, acute myocardial infarction, chronic ulcer of the skin and urinary incontinence were associated with 3.42, 1.65, and 1.35 times higher mortality. Corticosteroids, vasodilators, immunosuppressants, and antipsychotics were amongst the medications associated the most with an increased likelihood of mortality. The models explained 22.8% and 25.9% of the variance of mortality in men and women, respectively.

## 4. Discussion

In this study, we described the main socio-demographic and baseline clinical characteristics of the first 771 deceased individuals with laboratory-confirmed SARS-CoV-2 infection in Aragon, Spain. We analyzed which factors were associated with an increased likelihood of mortality in order to identify potential profiles of men and women to whom prevention and control strategies should be directed during the present and possible future outbreaks of the COVID-19 disease. Our findings suggest that women and men probably share similar but not identical mortality risk factors, and that the focus should be set on older individuals with cardiovascular comorbidity and under treatment with high anticholinergic activity medications, like antipsychotics.

Advanced age is associated with a higher risk of mortality in COVID-19 patients [11,18], which concurs with our findings (more markedly in women). We believe that the higher mortality rates reported in our study, especially among 80-year-old patients and over, could be partially explained by the severity of their clinical status (laboratory testing for COVID-19 infection was initially implemented only in patients at high risk of adverse outcomes), and the large follow-up period (up to ten weeks and at least one month from cohort entry) which allowed for a higher number of definite outcomes to occur. A recent study in 244 hospitalized COVID-19 patients aged 60 and over, with definite outcomes (discharged or deceased), reported 121 deaths (mortality rate of approximately 50%), identifying older age as one of the most important risk factors [19]. In Spain, the pandemic hit hardest in residential care homes for the elderly, at least during the first weeks of the crisis when less was known about the disease´s transmissibility and pathophysiology. This could explain, in part, the older age of infected patients in our study when compared to those of other regions. Individuals living in residential care homes are amongst the most susceptible to poorer health outcomes from COVID-19, mainly due to the higher number of underlying chronic conditions, immunosenescence, and the potential for rapid transmissibility from asymptomatic and pre-symptomatic patients [20,21,22]. Although the infection was more prevalent in women, we found that COVID-19 mortality was more common in men. In female adults, innate and adaptive immunity is generally stronger than in men, and males are more susceptible to infectious diseases [23]. A closer look at the deceased population in our study reveals that men were younger than women, with a lower mean number of comorbidities and medication profile with lower anticholinergic activity. A higher mean number of medications appears to be associated with poorer outcomes, especially in drugs with anticholinergic activity.

During seasonal influenza outbreaks, the reported incidence of acute cardiovascular events in elders traditionally grows, and emergency-visits for influenza-like disease have been associated with cardiovascular morbidity and mortality [24]. Previous studies reported that some patients with COVID-19 develop acute cardiac injury and that this comorbidity may result in a poorer prognosis [9,25]. It has also been suggested that patients with underlying cardiovascular conditions might also have an increased risk of adverse outcomes [26,27]. Our results show that chronic cardiovascular comorbidity in COVID-19 patients is associated with an increased likelihood of mortality. More precisely, male COVID-19 patients with non-hypertensive congestive heart failure are more likely to die than men without this condition. In female COVID-19 patients, we observe similar results for clinical history of acute myocardial infarction or cardiac dysrhythmias. These findings underline the need to understand how SARS-CoV-2 interacts with cardiovascular comorbidities and medications. Regarding the recent controversy over a possible adverse effect of seasonal flu shot on COVID-19 prognosis, we must highlight that no association between influenza vaccination and risk of mortality was found in our study.

Viral respiratory infections could result in the activation of pathways leading to vascular complications [24]. Various mechanisms could be contributing to cardiac injury by SARS-CoV-2, including, amongst others, direct viral infection of myocardial cells mediated by angiotensin-converting enzyme 2 (ACE2) receptors [25,26,27]. Activation of coagulation and inflammatory pathways could lead to vascular complications, theoretically both in the form of newly presented acute cardiovascular diseases or the deterioration of underlying chronic conditions. Various authors have recommended caution when prescribing ACE inhibitors or angiotensin-receptor blockers (ARBs) in COVID-19 patients with cardiovascular comorbidity, as this medication can increase ACE2 levels, which could potentially lead to a more severe health outcomes [28,29]. However, in our study, we did not observe any association of plain ACE inhibitors or ARBs with mortality. This concurs with other authors, who did not find evidence of an increased risk of adverse outcomes in patients treated with ACE2 and ARBs in their studies [30]; thus, we believe that more research is needed on this aspect. Our findings underscore the importance of studying the role of ACE2 receptors in the pathophysiology of COVID-19 and of conducting longitudinal population-based epidemiological studies in order to design and validate proper preventive strategies for patients at high risk of adverse outcomes.

Multimorbidity and polypharmacy are very common among the elderly, making it challenging to identify and interpret all the possible interactions among diseases and/or drugs, especially in the presence of an emerging disease such as this one. An interesting finding in our study was that the association of specific pharmacological subgroups with a higher likelihood of mortality does not necessarily constitute a therapeutic indication for chronic diseases that were also identified as potential risk factors. This observation should be interpreted with caution, as there are many epidemiological, clinical, and pathophysiological factors that could contribute to this finding. Possible drug-related side effects and adverse reactions, and drug–drug or drug–disease interactions, could result in a substantially higher risk of poorer prognosis, particularly in multimorbid patients. Our findings may provide useful information for future studies on this aspect. An interesting observation was the higher likelihood of mortality in patients who were in treatment with high anticholinergic activity medications. Potential adverse consequences of such treatments in the elderly include cognitive and functional decline, cardiovascular complications, and other adverse effects that could lead to institutionalization or other serious outcomes [31,32,33].

By analyzing all statistically significant associations with an increased likelihood of mortality, we identified different models for men and women. Although these models might describe patients with different clinical profiles, a common and constant characteristic is the presence of diseases that could originate/cause vascular complications, or of medications prescribed for such conditions. Nonetheless, we must highlight that these models explained up to 23% and 26% of the variance of mortality in men and women, respectively. This means that approximately 75% of the mortality would be explained by other factors probably related to genetics, other clinical variables, or the management of the patient. Furthermore, statistical associations do not necessarily translate into the existence of causal relationships, and these findings should be interpreted with caution. Future large-scale population studies based on real-world data in different European settings are encouraged to provide the necessary evidence for the development of early identification strategies for patients at higher risk of adverse outcomes.

The main strength of this study lies in the fact that we analyzed all the individuals of the reference population with a laboratory-confirmed infection by COVID-19 who died during the study period. Baseline comorbidity was individually and exhaustively studied by analyzing a list of 153 chronic conditions, and not only the most common or severe ones. The medication burden was also exhaustively analyzed, however, we only focused on medications dispensed during the month prior to the infection, and we might have missed some of the patients’ treatments. Another limitation lies in the unavailability of certain variables that would have been of interest for the aim of our study, such as socio-economic variables, genetics, laboratory tests, and inpatient treatments, amongst others.

## 5. Conclusions

Our findings suggest that the comorbidity and medication burden present at infection with SARS-CoV-2 could partially affect the outcome of the COVID-19 disease, and that women and men probably share similar but not identical risk factors. Advanced age seems to be the most influencing factor, followed by the presence of various underlying conditions and medications, particularly those related to the cardiovascular system. Nonetheless, much of the mortality seems to be explained by other factors probably related to patients´ clinical management, genetics, and other clinical variables. More research is needed in this field, in different populations and clinical settings, to provide the necessary evidence for the development of early identification strategies for patients at higher risk of adverse outcomes.

## Figures and Tables

**Table 1:** Socio-demographic and clinical characteristics at cohort entry of the individuals with laboratory-confirmed infection by COVID-19 in Aragón, Spain, March to May 2020 (*n* = 4412), by sex.

Characteristics	Alive ^a^ (*n* = 3641)				Dead (*n* = 771)				*p* Value ^b^	
	Men		Women		Men		Women		Men	Women
	*n*	%	*n*	%	*n*	%	*n*	%		
	1414	38.8	2229	61.2	407	52.8	364	47.2		
**Age**, years										
0–14	6	0.42	0	0	0	0	0	0	-	-
15–44	205	14.5	516	23.1	1	0.25	0	0	0.019	-
45–64	540	38.2	844	37.9	29	7.13	7	1.92	Reference	Reference
65–79	352	24.9	318	14.3	121	29.7	51	14.0	<0.001	<0.001
≥80	309	21.9	551	24.7	256	62.9	306	84.1	<0.001	<0.001
Mean (mean, sd ^c^)	63.3	18.1	60.8	20.2	81.5	10.7	87.2	8.20	<0.001	<0.001
**Non-native** ^d^	163	11.5	299	13.4	8	1.97	7	1.92	0.369	0.649
**Rural area** ^e^	510	36.1	768	34.5	168	41.3	161	44.2	0.121	0.473
**Deprivation index** ^f^										
Q1	474	33.6	743	33.3	130	31.9	142	39.0	Reference	Reference
Q2	339	24	513	23.0	96	23.6	78	21.4	0.156	0.657
Q3	275	19.5	423	19.0	87	21.4	70	19.2	0.729	0.078
Q4	324	22.9	550	24.7	94	23.1	74	20.3	0.458	0.849
**Flu vaccination**, yes	512	36.3	710	31.9	261	64.1	249	68.4	0.110	0.126
**No. of chronic diseases**^g^ (mean, sd)	3.03	3.35	3.67	3.65	5.22	4.02	6.66	4.14	0.012	0.003
**Multimorbidity** ^h^	793	56.2	1410	63.3	307	75.4	311	85.4	0.398	0.022
**No. of drugs**^i^ (mean, sd)	3.00	3.14	2.75	3.11	5.41	3.73	5.57	3.42	<0.001	0.006
**Polypharmacy** ^j^	402	28.5	568	25.5	242	59.5	217	59.6	<0.001	0.125
**ADS score** ^k^										
0	1106	78.3	1680	75.4	234	57.5	168	46.2	Reference	Reference
1	152	10.8	270	12.1	76	18.7	85	23.4	0.088	0.044
2	79	5.59	128	5.74	47	11.5	60	16.5	0.091	0.003
≥3	75	5.31	151	6.77	50	12.3	51	14.0	0.010	0.070

^a^ Alive or dead at the end of the follow-up period; ^b^ *p*-value of the comparison between alive and dead individuals by sex, using age-adjusted logistic regression analysis; ^c^ standard deviation; ^d^ Born out of Spain; ^e^ Based on the type of administrative health area (urban, if 80% or more of the population of the area is concentrated in one of the municipalities, or rural for the rest); ^f^ Calculated at aggregated level per administrative health area and divided into quartiles from less (Q1) to more (Q4) deprived; ^g^ Primary care diagnoses from a list of 153 chronic conditions, using the Clinical Classification System and the Chronic Condition Indicator; ^h^ Defined as the presence of two or more chronic diseases; ^i^ Dispensed during the month prior to cohort entry, using the Anatomical-Therapeutic-Chemical classification system at the third level (i.e., pharmacological subgroup); ^j^ Defined as the dispensation of five or more drugs; ^k^ Anticholinergic Drug Scale score.

**Table 2:** Prevalence of chronic diseases at cohort entry of individuals that died during the follow-up period, and association with all-cause mortality, by sex.

Chronic Conditions	Men (*n* = 407)			95% CI ^a^		Women (*n* = 364)			95% CI	
CCS Code ^b^	*n*	%	OR ^c^	Lower Limit	Upper Limit	*n*	%	OR	Lower Limit	Upper Limit
Hypertension	201	49.4	0.96	0.75	1.23	228	62.6	1.20	0.92	1.56
**Urinary incontinence**	103	25.3	1.33	0.97	1.83	184	50.5	**1.50**	**1.16**	**1.94**
Obesity and other nutritional, endocrine, metabolic dis. ^d^	117	28.7	1.02	0.78	1.34	138	37.9	1.26	0.97	1.63
**Cataract**	114	28.0	**1.62**	**1.20**	**2.18**	99	27.2	1.19	0.89	1.59
**Mood disorders**	70	17.2	1.38	0.98	1.95	132	36.3	**1.46**	**1.12**	**1.91**
Osteoarthritis	74	18.2	0.93	0.67	1.29	121	33.2	1.00	0.77	1.31
**Diabetes Mellitus**	112	27.5	**1.65**	**1.23**	**2.21**	80	22.0	**1.40**	**1.02**	**1.91**
Delirium, dementia	60	14.7	1.04	0.72	1.49	101	27.7	1.20	0.90	1.61
Dis. of lipid metabolism	70	17.2	1.22	0.88	1.70	61	16.8	0.92	0.66	1.28
Anxiety dis.	38	9.34	0.80	0.53	1.21	83	22.8	1.21	0.90	1.64
Chronic renal failure	55	13.5	1.32	0.89	1.94	61	16.8	1.24	0.87	1.76
**Cardiac dysrhythmias**	58	14.3	1.01	0.70	1.46	58	15.9	**1.47**	**1.02**	**2.12**
Osteoporosis	9	2.21	1.16	0.48	2.82	95	26.1	1.27	0.95	1.70
Other ear/sense dis.	54	13.3	0.85	0.59	1.22	53	14.6	0.90	0.63	1.28
Spondylosis	45	11.1	1.27	0.84	1.92	54	14.8	1.10	0.77	1.57
Hyperplasia of prostate	97	23.8	1.15	0.86	1.56	0	0	-	-	-
**Congestive heart failure**	41	10.1	**2.15**	**1.30**	**3.54**	40	11.0	1.40	0.91	2.15
**Chronic ulcer of skin**	30	7.37	**2.24**	**1.26**	**3.96**	50	13.7	**2.04**	**1.35**	**3.10**
Thyroid dis.	26	6.39	1.18	0.70	1.97	53	14.6	0.82	0.58	1.16
Glaucoma	39	9.58	0.69	0.46	1.05	41	11.3	1.05	0.70	1.57
**Hereditary/degenerative nervous system conditions**	23	5.65	1.43	0.79	2.60	49	13.5	**1.56**	**1.05**	**2.33**
**Blindness and vision defects**	48	11.8	**1.70**	**1.12**	**2.57**	19	5.22	0.78	0.46	1.35
**Coagulation/hemorrhagic dis.**	29	7.13	0.84	0.52	1.36	35	9.62	**1.85**	**1.15**	**2.97**
Headache, including migraine	20	4.91	0.64	0.38	1.09	43	11.8	0.94	0.64	1.38
**Acute cerebrovascular dis.**	34	8.35	**1.83**	**1.09**	**3.07**	29	7.97	1.26	0.77	2.06
Transient cerebral ischemia	30	7.37	1.60	0.93	2.73	26	7.14	0.86	0.53	1.41
Neoplasms	31	7.62	1.22	0.75	1.98	18	4.95	1.41	0.77	2.58
COPD ^e^ and bronchiectasis	38	9.34	0.99	0.64	1.52	10	2.75	1.17	0.54	2.55
Upper respiratory disease	23	5.65	1.29	0.75	2.21	19	5.22	1.04	0.60	1.81
Coronary atherosclerosis	24	5.90	1.05	0.62	1.81	17	4.67	1.07	0.59	1.97
Diverticulosis/diverticulitis	18	4.42	1.25	0.67	2.35	22	6.04	1.25	0.72	2.16
Gout	30	7.37	0.85	0.54	1.35	10	2.75	1.57	0.68	3.61
**Acute myocardial infarction**	22	5.41	1.53	0.85	2.73	13	3.57	**2.98**	**1.25**	**7.11**
Miscellaneous mental health dis.	14	3.44	0.64	0.34	1.19	19	5.22	1.38	0.77	2.46
Other nervous system dis.	16	3.93	0.87	0.46	1.62	15	4.12	0.79	0.43	1.44
Acquired foot deformities	5	1.23	0.91	0.30	2.74	24	6.59	0.82	0.50	1.34
Developmental dis.	11	2.70	1.32	0.60	2.92	16	4.40	1.22	0.65	2.27
Parkinson’s disease	13	3.19	1.63	0.73	3.67	13	3.57	2.10	0.96	4.60
Asthma	6	1.47	0.45	0.18	1.11	19	5.22	0.68	0.40	1.17
Prolapse of genital organs	0	0	-	-	-	24	6.59	1.53	0.89	2.61
Other non-traumatic joint dis.	10	2.46	0.90	0.40	2.03	15	4.12	0.89	0.48	1.66
Retinal detachments, vascular occlusion, retinopathy	12	2.95	1.89	0.84	4.25	13	3.57	1.15	0.58	2.29
Residual codes; unclassified	18	4.42	1.44	0.78	2.66	5	1.37	1.84	0.58	5.84
Menopausal dis.	0	0	-	-	-	20	5.49	0.97	0.57	1.66
Other gastrointestinal dis.	11	2.70	1.66	0.71	3.88	11	3.02	1.04	0.50	2.14
Allergic reactions	14	3.44	2.02	0.93	4.39	8	2.20	0.72	0.32	1.64
Other inflammatory condition skin	15	3.69	1.74	0.86	3.53	7	1.92	0.75	0.31	1.79
Esophageal dis.	9	2.21	0.80	0.37	1.73	11	3.02	0.85	0.42	1.72
Other endocrine dis.	9	2.21	1.23	0.50	3.05	11	3.02	1.92	0.84	4.38
**Epilepsy, convulsions**	12	2.95	**2.56**	**1.09**	**5.99**	8	2.20	0.96	0.41	2.25
Adjustment dis.	6	1.47	0.72	0.27	1.92	13	3.57	0.98	0.50	1.92
Peripheral, visceral atherosclerosis	16	3.93	1.79	0.86	3.73	2	0.55	0.96	0.18	5.15
Menstrual dis.	0	0	-	-	-	14	3.85	1.31	0.68	2.53
**Inflammatory conditions of male genital organs**	13	3.19	**2.56**	**1.15**	**5.70**	0	0	-	-	-
Other bone disease and musculoskeletal deformities	1	0.25	0.38	0.04	3.40	10	2.75	1.47	0.65	3.31
Other male genital dis.	10	2.46	1.41	0.65	3.06	0	0	-	-	-

^a^ Confidence interval; ^b^ Primary care diagnoses from a list of 153 chronic conditions, using the Clinical Classification System (CCS) and the Chronic Condition Indicator. Diseases are listed in order of descending overall prevalence; only those with at least 10 cases in men and/or women are presented. The complete list of diseases is presented in Supplementary Table S1; ^c^ Odds ratio; the OR of mortality for each basal chronic disease was estimated using logistic regression models. The ORs were adjusted by age, included in the model as a categorical variable. Conditions with statistically significant ORs in men and/or women are highlighted in bold. All the individuals of the cohort were followed for at least 30 days after cohort entry; ^d^ Disorders; ^e^ Chronic obstructive pulmonary disease.

**Table 3:** Prevalence of medications dispensed during the month prior to cohort entry of individuals that died during the follow-up period, and association with all-cause mortality, by sex.

Medications	Men (*n* = 407)			95% CI ^a^		Women (*n* = 364)			95% CI	
ATC Code ^b^	*n*	%	OR ^c^	Lower Limit	Upper Limit	*n*	%	OR	Lower Limit	Upper Limit
**Drugs for peptic ulcer, GORD ^d^ (A02B)**	202	49.6	**1.45**	**1.13**	**1.86**	213	58.5	**1.32**	**1.02**	**1.71**
**Antithrombotic agents (B01A)**	150	36.9	1.25	0.96	1.63	142	39.0	**1.51**	**1.16**	**1.97**
Other analgesics and antipyretics (N02B)	126	31.0	1.04	0.80	1.36	136	37.4	0.93	0.72	1.21
**High-ceiling diuretics** **(C03C)**	100	24.6	**1.79**	**1.30**	**2.49**	126	34.6	**1.71**	**1.29**	**2.27**
**Antidepressants (N06A)**	83	20.4	1.30	0.94	1.79	141	38.7	**1.33**	**1.02**	**1.73**
Lipid modifying agents, plain (C10A)	93	22.9	0.93	0.69	1.24	70	19.2	0.83	0.60	1.13
**Beta blocking agents (C07A)**	85	20.9	**1.53**	**1.11**	**2.11**	68	18.7	1.28	0.92	1.79
**Antipsychotics (N05A)**	70	17.2	**1.71**	**1.18**	**2.48**	82	22.5	**1.96**	**1.41**	**2.73**
**Anxiolytics (N05B)**	57	14.0	**1.52**	**1.05**	**2.22**	89	24.5	1.32	0.98	1.78
Blood glucose lowering drugs (A10B)	73	17.9	1.14	0.82	1.58	48	13.2	1.28	0.88	1.88
Drugs for prostatic hypertrophy (G04C)	108	26.5	0.96	0.72	1.28	0	0	-	-	-
Opioids (N02A)	42	10.3	1.13	0.75	1.71	62	17.0	1.30	0.92	1.83
**Antiepileptics (N03A)**	37	9.09	1.00	0.65	1.55	52	14.3	**1.94**	**1.31**	**2.88**
ARBs ^e^, plain (C09C)	45	11.1	1.16	0.78	1.74	42	11.5	0.99	0.67	1.46
ARBs, comb. ^f^ (C09D)	49	12.0	1.00	0.69	1.46	32	8.79	0.68	0.44	1.03
ACE ^g^ inhibitors, plain (C09A)	40	9.83	0.90	0.60	1.36	38	10.4	1.14	0.74	1.73
Iron preparations (B03A)	35	8.60	1.44	0.88	2.35	38	10.4	1.06	0.70	1.62
**Vitamin B12 and folic acid (B03B)**	45	11.1	**1.88**	**1.20**	**2.97**	28	7.69	1.16	0.71	1.88
Adrenergics, inhalants (R03A)	45	11.1	1.35	0.90	2.02	24	6.59	0.98	0.60	1.62
Selective calcium channel blockers with mainly vascular effects (C08C)	39	9.58	1.10	0.72	1.69	27	7.42	0.82	0.51	1.31
Hypnotics and sedatives (N05C)	30	7.37	1.04	0.64	1.69	36	9.89	0.92	0.60	1.39
Antiglaucoma preparations (S01E)	33	8.11	1.02	0.64	1.61	25	6.87	1.07	0.65	1.76
**Antigout preparations (M04A)**	35	8.60	1.29	0.82	2.04	17	4.67	**2.06**	**1.05**	**4.04**
Anti-dementia drugs (N06D)	18	4.42	0.73	0.40	1.32	33	9.07	0.92	0.60	1.42
Macrolides, lincosamides (J01F)	26	6.39	0.84	0.51	1.36	23	6.32	1.19	0.71	2.01
**Other drugs for obstructive airway diseases, inhalants (R03B)**	33	8.11	**1.83**	**1.10**	**3.05**	16	4.40	0.96	0.52	1.76
B-lactam antibacterials, penicillins (J01C)	22	5.41	0.84	0.49	1.42	24	6.59	1.62	0.94	2.79
Quinolone antibacterials (J01M)	31	7.62	1.55	0.94	2.56	15	4.12	1.37	0.70	2.66
**Potassium-sparing agents (C03D)**	23	5.65	**2.52**	**1.27**	**4.97**	23	6.32	**2.86**	**1.50**	**5.44**
Corticosteroids systemic use, plain (H02A)	23	5.65	1.44	0.82	2.54	21	5.77	1.13	0.65	1.94
**Insulins and analogues (A10A)**	21	5.16	**2.33**	**1.21**	**4.48**	16	4.40	1.40	0.73	2.68
**Vasodilators for cardiac diseases (C01D)**	24	5.90	**3.15**	**1.55**	**6.41**	12	3.30	1.15	0.55	2.40
Calcium (A12A)	12	2.95	1.69	0.73	3.88	23	6.32	0.70	0.43	1.15
Other systemic drugs for obstructive airway diseases (R03D)	20	4.91	1.18	0.66	2.11	10	2.75	0.78	0.37	1.62
Vitamin A and D (A11C)	7	1.72	0.90	0.35	2.30	21	5.77	1.73	0.97	3.10
**Other beta-lactam antibacterials (J01D)**	15	3.69	2.02	0.95	4.30	13	3.57	**2.17**	**1.00**	**4.71**
Anti-inflammatory/ antirheumatic products, non-steroids (M01A)	13	3.19	0.58	0.31	1.10	13	3.57	0.70	0.37	1.32
*ACE inhibitors, comb. (C09B)*	11	2.70	*0.46*	*0.23*	*0.93*	15	4.12	1.00	0.53	1.87
Other antibacterials (J01X)	8	1.97	0.80	0.34	1.91	18	4.95	1.17	0.64	2.13
Antihistamines for systemic use (R06A)	15	3.69	1.46	0.74	2.88	10	2.75	1.54	0.70	3.40
Dopaminergic agents (N04B)	17	4.18	1.15	0.60	2.21	8	2.20	1.17	0.48	2.81
Urologicals (G04B)	17	4.18	1.34	0.69	2.62	7	1.92	0.80	0.34	1.92
Psychostimulants, agents used for ADHD ^h^ and nootropics (N06B)	14	3.44	2.01	0.89	4.54	9	2.47	1.24	0.54	2.89
Thyroid preparations (H03B)	8	1.97	1.22	0.48	3.09	14	3.85	0.98	0.52	1.86
Drugs affecting bone structure and mineralization (M05B)	7	1.72	2.42	0.71	8.22	14	3.85	0.82	0.43	1.56
Selective calcium channel blockers with direct cardiac effects (C08D)	11	2.70	1.09	0.50	2.37	10	2.75	1.46	0.65	3.31
**Immunosuppressants (L04A)**	11	2.70	**2.49**	**1.08**	**5.76**	3	0.82	0.89	0.24	3.27
**Corticosteroids, plain (D07A)**	11	2.70	**3.83**	**1.43**	**10.3**	3	0.82	0.61	0.16	2.25
Lipid modifying agents, comb. (C10B)	10	2.46	0.95	0.44	2.02	2	0.55	0.29	0.07	1.27

^a^ Confidence interval; ^b^ Drugs were coded using the Anatomical-Therapeutic-Chemical (ATC) classification system at the third level (i.e., pharmacological subgroup). Drugs are listed in descending order of overall frequency of dispensation; only those with at least 10 cases in men and/or women are presented. The complete list of drugs is presented in Supplementary Table S2; ^c^ Odds ratio; the OR of mortality for each dispensed drug was estimated using logistic regression models. The ORs were adjusted by age, included in the model as a categorical variable. Drugs with statistically significant ORs in men and/or women are highlighted in bold (OR > 1) or in italics (OR < 1). All the individuals of the cohort were followed for at least 30 days after cohort entry; ^d^ Gastro-esophageal reflux disease; ^e^ Angiotensin II receptor blockers; ^f^ Combinations; ^g^ Angiotensin-converting enzyme; ^h^ Attention-deficit hyperactivity disorder.

**Table 4:** Models of likelihood of mortality according to baseline demographic and clinical variables of the individuals with laboratory-confirmed infection by COVID-19, by sex.

Variables ^a^	OR ^b^	95% CI ^c^ Lower Limit	95% CI Upper Limit	*p*-Value
**Men** (*n* = 407)				
*Age (years)*				
0–14	-			
15–44	0.10	0.01	0.73	0.024
45–64	Reference			
65–79	5.18	3.35	8.03	<0.001
≥80	10.4	6.75	16.1	<0.001
*Chronic diseases*				
Chronic ulcer of skin	2.04	1.14	3.65	0.017
Cataract	1.48	1.08	2.01	0.014
Diabetes Mellitus	1.46	1.08	1.98	0.014
*Drugs*				
Corticosteroids, single drugs	3.32	1.25	8.80	0.016
Vasodilators used in cardiac diseases	2.77	1.33	5.75	0.006
Immunosuppressive agents	2.54	1.08	5.98	0.033
Potassium-sparing agents	2.45	1.23	4.88	0.011
Antipsychotics	1.66	1.13	2.43	0.009
Vitamin B12 and folic acid	1.69	1.05	2.70	0.029
**Women** (*n* = 364)				
*Age (years)*				
0–14	-			
15–44	-			
45–64	Reference			
65–79	15.7	6.99	35.0	0.000
≥80	40.7	18.8	88.3	0.000
*Chronic diseases*				
Acute myocardial infarction	3.42	1.41	8.32	0.007
Chronic ulcer of skin	1.65	1.07	2.54	0.024
Urinary incontinence	1.35	1.04	1.77	0.027
*Drugs*				
Peripheral vasodilators	5.49	1.29	23.3	0.021
Potassium-sparing agents	2.17	1.10	4.29	0.026
Antipsychotics	1.81	1.29	2.53	0.001
Antiepileptics	1.63	1.08	2.45	0.019
High-ceiling diuretics	1.41	1.04	1.90	0.028

^a^ Demographic (age) and clinical (chronic diseases and drugs) variables that remained significant of all the analyzed ones in each of the models after performing multivariate stepwise logistic regression with an exit *p*-value for variables of 0.05; ^b^ Odds ratio; ^c^ Confidence interval.

## Supplementary Materials

The following are available online at https://www.mdpi.com/1660-4601/17/14/5171/s1, Table S1: Prevalence of chronic diseases at cohort entry of all the individuals of the COVID-19 Aragon cohort and of those died during the follow-up period, and association with mortality, by sex; Table S2: Prevalence of drugs dispensed during the month prior to cohort entry of all the individuals of the COVID-19 Aragon cohort and of those died during the follow-up period, and association with mortality, by sex.

## Appendix A

Other investigators of the EpiChron Research Group on Chronic Diseases: Mercedes Clerencia-Sierra (Miguel Servet University Hospital, Aragon Health Service, Zaragoza, Spain), Carlos Coscollar-Santaliestra (San Pablo Primary Care Health Centre, Aragon Health Service, Zaragoza, Spain), Inmaculada Guerrero-Fernández de Alba (Teaching Unit of Preventive Medicine and Public Health, Miguel Servet University Hospital, Zaragoza, Spain), Aida Moreno-Juste (Aragon Health Service, Zaragoza, Spain), Victoria Pico-Soler (Torrero-La Paz Primary Care Health Centre, Aragon Health Service, Zaragoza, Spain), and Paula Ara-Bardají (Aragon Institute for Health Research (IIS Aragón).

## References

[1] Li Q., Guan X., Wu P., Wang X., Zhou L., Tong Y., Ren R., Leung K.S., Lau E.H., Wong J.Y. (2020). Early Transmission Dynamics in Wuhan, China, of Novel Coronavirus—Infected Pneumonia. N. Engl. J. Med..

[2] Chan J.F.-W., Yuan S., Kok K.-H., To K.K.-W., Chu H., Yang J., Xing F., Liu J., Yip C.C.-Y., Poon R.W.-S. (2020). A familial cluster of pneumonia associated with the 2019 novel coronavirus indicating person-to-person transmission: A study of a family cluster. Lancet.

[3] WHO Coronavirus Disease (COVID-19) Dashboard. https://covid19.who.int/.

[4] Pareek M., Bangash M.N., Pareek N., Pan D., Sze S., Minhas J.S., Hanif W., Khunti K. (2020). Ethnicity and COVID-19: An urgent public health research priority. Lancet.

[5] Zhao H., Harris R.J., Ellis J., Pebody R.G. (2015). Ethnicity, deprivation and mortality due to 2009 pandemic influenza A (H1N1) in England during the 2009/2010 pandemic and the first post-pandemic season. Epidemiol. Infect..

[6] Weiss P., Murdoch D.R. (2020). Clinical course and mortality risk of severe COVID-19. Lancet.

[7] Chen N., Zhou M., Dong X., Qu J., Gong F., Han Y., Qiu Y., Wang J., Liu Y., Wei Y. (2020). Epidemiological and Clinical Characteristics of 99 Cases of 2019—Novel Coronavirus (2019-nCoV) Pneumonia in Wuhan, China: A descriptive. SSRN Electron. J..

[8] Wu Z., McGoogan J.M. (2020). Characteristics of and Important Lessons from the Coronavirus Disease 2019 (COVID-19) Outbreak in China: Summary of a Report of 72314 Cases from the Chinese Center for Disease Control and Prevention. JAMA.

[9] Huang C., Wang Y., Li X., Ren L., Zhao J., Hu Y., Zhang L., Fan G., Xu J., Gu X. (2020). Clinical features of patients infected with 2019 novel coronavirus in Wuhan, China. Lancet.

[10] Guan W.-J., Liang W.-H., Zhao Y., Liang H.-R., Chen Z.-S., Li Y.-M., Liu X.-Q., Chen R.-C., Tang C.-L., Wang T. (2020). Comorbidity and its impact on 1590 patients with COVID-19 in China: A nationwide analysis. Eur. Respir. J..

[11] Zhou F., Yu T., Du R., Fan G., Liu Y., Liu Z., Xiang J., Wang Y., Song B., Gu X. (2020). Clinical course and risk factors for mortality of adult inpatients with COVID-19 in Wuhan, China: A retrospective cohort study. Lancet.

[12] Dea M.L.C., Bellido E.O., Solana C.F., Palacio I.A., Del Hombrebueno G.G.-C.R., Sancho B.A. (2018). Construction of a deprivation index by Basic Healthcare Area in Aragon using Population and Housing Census 2011. Rev. Esp. Salud Publica.

[13] Carnahan R., Lund B.C., Perry L., Pollock B.G., Culp K.R. (2006). The Anticholinergic Drug Scale as a Measure of Drug-Related Anticholinergic Burden: Associations with Serum Anticholinergic Activity. J. Clin. Pharmacol..

[14] Eum S., Hill S.K., Rubin L.H., Carnahan R.M., Reilly J.L., Ivleva E.I., Sarah K., Carol A., Godfrey D., Brett A. (2017). Cognitive burden of anticholinergic medications in psychotic disorders. Schizophr. Res..

[15] Ancín Ducay J.M., Erce López S., Extramiana Cameno E., Izcue Argandoña A. (2014). Correlación de Códigos CIE-9-MC (8a Edic.)—CIAP-2 Para la Gestión de Incapacidad Temporal.

[16] Elixhauser A., Steiner C., Palmer L. Clinical Classifications Software (CCS), 2009. Agency for Healthcare Research and Quality. http://www.hcup-us.ahrq.gov/toolssoftware/ccs/ccs.jsp.

[17] Chronic Condition Indicator (CCI) for ICD-9-CM. https://www.hcup-us.ahrq.gov/toolssoftware/chronic/chronic.jsp.

[18] Onder G., Rezza G., Brusaferro S. (2020). Case-Fatality Rate and Characteristics of Patients Dying in Relation to COVID-19 in Italy. JAMA.

[19] Sun H., Ning R., Tao Y., Yu C., Deng X., Zhao C., Meng S., Tang F., Xu D. (2020). Risk Factors for Mortality in 244 Older Adults With COVID-19 in Wuhan, China: A Retrospective Study. J. Am. Geriatr. Soc..

[20] American Geriatrics Society (2020). American Geriatrics Society Policy Brief: COVID-19 and Nursing Homes. J. Am. Geriatr. Soc..

[21] D’Adamo H., Yoshikawa T., Ouslander J.G. (2020). Coronavirus Disease 2019 in Geriatrics and Long-Term Care: The ABCDs of COVID-19. J. Am. Geriatr. Soc..

[22] Arons M.M., Hatfield K.M., Reddy S.C., Kimball A., James A., Jacobs J.R., Taylor J., Spicer K., Bardossy A.C., Oakley L.P. (2020). Presymptomatic SARS-CoV-2 Infections and Transmission in a Skilled Nursing Facility. N. Engl. J. Med..

[23] Klein S.L., Flanagan K.L. (2016). Sex differences in immune responses. Nat. Rev. Immunol..

[24] Nguyen J.L., Yang W., Ito K., Matte T.D., Shaman J., Kinney P.L. (2016). Seasonal Influenza Infections and Cardiovascular Disease Mortality. JAMA Cardiol..

[25] Shi S., Qin M., Shen B., Cai Y., Liu T., Yang F., Gong W., Liu X., Liang J., Zhao Q. (2020). Association of Cardiac Injury With Mortality in Hospitalized Patients With COVID-19 in Wuhan, China. JAMA Cardiol..

[26] Zheng Y.Y., Ma Y.T., Zhang J.Y., Xie X. (2020). COVID-19 and the cardiovascular system. Nat. Rev. Cardiol..

[27] Zhu H., Rhee J.W., Cheng P., Waliany S., Chang A., Witteles R.M., Maecker H., Davis M.M., Nguyen P.K., Wu S.M. (2020). Cardiovascular Complications in Patients with COVID-19: Consequences of Viral Toxicities and Host Immune Response. Curr. Cardiol. Rep..

[28] Guan W.-J., Ni Z.-Y., Hu Y., Liang W.-H., Ou C.-Q., He J.-X., Liu L., Shan H., Lei C.-L., Hui D.S. (2020). Clinical Characteristics of Coronavirus Disease 2019 in China. N. Engl. J. Med..

[29] Fang L., Karakiulakis G., Roth M. (2020). Are patients with hypertension and diabetes mellitus at increased risk for COVID-19 infection?. Lancet Respir. Med..

[30] Li J., Wang X., Chen J., Zhang H., Deng A. (2020). Association of Renin-Angiotensin System Inhibitors With Severity or Risk of Death in Patients with Hypertension Hospitalized for Coronavirus Disease 2019 (COVID-19) Infection in Wuhan, China. JAMA Cardiol..

[31] Singh S., Loke Y.K., Furberg C.D. (2008). Inhaled Anticholinergics and Risk of Major Adverse Cardiovascular Events in Patients with Chronic Obstructive Pulmonary Disease: A Systematic Review and Meta-analysis. JAMA.

[32] Salahudeen M.S., Duffull S., Nishtala P.S. (2015). Anticholinergic burden quantified by anticholinergic risk scales and adverse outcomes in older people: A systematic review. BMC Geriatr..

[33] Gamble D.T., Clark A.B., Luben R.N., Wareham N.J., Khaw K.-T., Myint P.K. (2018). Baseline anticholinergic burden from medications predicts incident fatal and non-fatal stroke in the EPIC-Norfolk general population. Int. J. Epidemiol..

